# Exploring the practices, challenges, and pathways for enhancement of grassroots social organizations in preventing child unintentional injuries: a qualitative study in Guizhou, China

**DOI:** 10.3389/fpubh.2026.1760634

**Published:** 2026-01-29

**Authors:** Xiujuan Li, Roumei Lin, Pan Wen, Liping Li, Linlin Xie, Yaogui Lu, Guanhua Fan, Zicheng Cao, Fancun Meng, Yanhong Huang, Jian-E Peng

**Affiliations:** 1 School of Public Health, Shantou University, Shantou, China; 2 Injury Prevention Research Center, Shantou University Medical College, Shantou, China; 3 Mental Health Center of Shantou University, Shantou, China; 4 College of Liberal Arts, Shantou University, Shantou, China

**Keywords:** left-behind children, unintentional injury, prevention and control practices, social organizations, qualitative study

## Abstract

**Background:**

Children’s unintentional injuries represent a global public health problem. Guizhou, as an economically underdeveloped province in southwest China, faces challenges in children’s unintentional injuries prevention and control, such as scarce resources and inadequate family supervision. Local social organizations play a crucial role in bridging the gap in government services. However, existing studies have predominantly focused on intervention models led by medical institutions or schools, lacking a systematic exploration of the practices and challenges of grassroots social organizations in this field.

**Objective:**

This study aims to explore the practices of grassroots social organizations in Guizhou Province regarding the prevention and control of children’s unintentional injuries, identify the internal and external challenges they face, and subsequently propose pathways for enhancement. The goal is to provide an evidence base for optimizing the governance of children’s safety at the grassroots level.

**Methods:**

This is a qualitative study. Using purposive sampling, semi-structured in-depth interviews were conducted in July 2024 with 15 social workers and 7 school administrators from two cities and one county in Guizhou Province. Thematic analysis of interview data was conducted using NVivo 15.

**Results:**

Currently, as frontline implementers, social organizations have developed various localized intervention strategies, such as experiential education and peer-led advocacy. Through collaboration with schools and communities—for instance, by constructing “grid-based management” and “full-time guardianship” models for left-behind children—they have achieved notable effects in enhancing children’s safety awareness and reducing injury incidents. However, the sustainability of these practices faces severe challenges. Internal challenges include insufficient human resources and funding and an imperfect training system. External challenges include poor cross-sectoral collaboration, a mismatch between interventions and children’s developmental stages, and weak family supervision. Based on this, participants suggested enhancing prevention and control practice by increasing resource investment, strengthening government-led collaborative mechanisms, and applying innovative technologies.

**Conclusion:**

Grassroots social organizations in Guizhou Province demonstrate localized innovative potential in the prevention and control of unintentional injury among children, but their effectiveness is constrained by systemic shortcomings. In the future, it is necessary to establish a multi-source resource support network, improve cross-sectoral collaboration mechanisms, and develop intervention strategies adapted to children’s cognitive characteristics.

## 1 Introduction

Unintentional injuries are a significant public health problem that threatens the health and lives of children worldwide (1). In China, injuries have become the leading cause of death among children and adolescents (2). Unintentional injuries among children are defined as external, sudden, and unintended events that cause harm to their health and life (3). The International Classification of Diseases, 10th Revision (ICD-10), categorizes unintentional injuries into ten types based on external causes of morbidity and mortality, including falls, road traffic injuries, drowning, and others (4). Although the National Program for Child Development (2021–2030) has explicitly outlined the injury prevention and control targets, significant implementation challenges persist at the grassroots level (5, 6).

Local social organizations play a critical role in addressing public health challenges, particularly in rural areas where government resources and services are limited. These organizations are often at the forefront of community health programs, leveraging their in-depth understanding of local needs and cultural customs to design intervention measures tailored to local conditions (7). Guizhou, as one of the economically underdeveloped provinces in southwest China, not only faces challenges such as high poverty rates and a scarcity of healthcare resources (8), but its prominent issue of “left-behind children” (9) has also resulted in a widespread and severe lack of family supervision. This specific dilemma renders traditional prevention and control models, which rely heavily on family supervision, largely ineffective. This, in turn, compels grassroots actors, such as social organizations, to explore new approaches and significantly complicates the prevention and control efforts.

There are still multiple limitations of current research on social organizations’ engagement in children’s unintentional injury prevention and control. A large body of research focuses on intervention models led by medical institutions and schools (10, 11), or their samples are primarily from more developed eastern regions (12), paying insufficient attention to the practical experiences of social organizations in underdeveloped western regions. Methodologically, most studies only measure quantitative indicators such as injury incidence rates (13, 14), failing to analyze the qualitative dimensions in depth. More importantly, in specific contexts like Guizhou, what specific localized intervention strategies have these social organizations developed in practice? What is the current state of their professional capacity as implementers? What challenges do they encounter in practice? And what is the sustainability of these strategies? These issues have not yet been systematically examined or analyzed.

Addressing this research gap, this study employed qualitative methodology to achieve three objectives: (1) to explore the practices of local social organizations in Guizhou Province regarding the prevention and control of children’s unintentional injuries; (2) to identify the internal and external challenges they face; and (3) to propose pathways for capability enhancement. The findings of this study can theoretically supplement the research on social organizations in western rural areas and also offer practical insights for the implementation of the China National Program for Child Development (2021–2030) in similar, underdeveloped regions.

## 2 Methods

### 2.1 Study design

This is a qualitative study. Semi-structured in-depth interviews were conducted between 8 and 22 July 2024 to collect data. The interview content focused on four core dimensions: (1) the cognition, prevention, and control status of children’s unintentional injury; (2) implementation difficulties and challenges; (3) effectiveness of preventive measures; (4) recommendations for prevention enhancement. The interview guide (see Supplementary File 1) was developed based on a comprehensive review of relevant literature and the specific research objectives. It was initially drafted by two researchers (X.L. and P.W.) with expertise in public health and qualitative methods. The draft was then reviewed and refined through iterative discussions and pilot-tested within the entire research team to ensure content validity and relevance to the local context.

### 2.2 Recruitment and participants

This study employed purposive sampling to recruit participants from Central Guizhou (Guiyang City), Southeast Guizhou (Liping County), and Southwest Guizhou (Xingyi City) in Guizhou Province. These sites were strategically selected to represent different levels of socioeconomic development and administrative hierarchies: Guiyang as the provincial capital with relatively abundant resources, Xingyi as a regional city, and Liping as a county-level area representing grassroots settings. This selection strategy allowed the study to capture diverse practices and challenges faced by social organizations across varying resource environments.

The recruitment followed the principle of identifying participants who possessed first-hand experience and deep insights into child unintentional injury interventions. Social workers were selected as the primary participants because, as frontline implementers, they could provide detailed accounts of intervention strategies, daily operations, and internal resource constraints. In total, 15 social workers were recruited. Preliminary fieldwork indicated that the prevention and control activities of social organizations are not conducted in isolation; rather, they are often carried out through a multi-sectoral collaboration model in which schools serve as primary partners. Consequently, in this study, seven school administrators were intentionally included as key informants to provide a comprehensive assessment of the collaboration, challenges, and overall practices of social organizations.

The inclusion criteria for participants were (1) full-time employees working in schools or grassroots social organizations in Guiyang City, Liping County, or Xingyi City, Guizhou Province; (2) their job responsibilities directly involved providing services to, or management for, children aged 3–12 years; and (3) able to communicate in Mandarin and agreed to participate in the interviews. The exclusion criteria were: (1) employees engaged only in administrative or logistical work, without direct involvement in child safety matters; (2) temporary staff or volunteers.

### 2.3 Data collection

Prior to the formal study, the interview guide was pilot-tested with three social workers and four school administrators to ensure clarity and relevance. Based on their feedback, minor adjustments were made to the wording to better align with local terminology.

The research team used telephone or WeChat for the recruitment process to explain the study purpose and schedule appointments. The formal semi-structured in-depth interviews were conducted face-to-face at the participants’ workplaces. To standardize the interview setting and ensure data quality, a strict protocol was followed. Researchers confirmed in advance that the interviews were held in quiet, private rooms to ensure confidentiality and allow participants to speak freely without being overheard.

Each interview lasted approximately 30–45 min. In addition to the main interviewer, one trained research assistant was present in each session to serve as a note-taker. The assistant was responsible for recording key nonverbal cues and monitoring the audio recording quality, ensuring no critical information was missed. All interviews were audio-recorded with participants’ consent.

### 2.4 Data analysis

We conducted thematic analysis using the constant comparative method (15). Adopting an inductive approach, themes were allowed to emerge directly from the data rather than being fitted into a pre-existing theoretical framework. All interviews were transcribed verbatim. Data collection and analysis were conducted concurrently to iteratively refine subsequent interviews. The coding and theme development process occurred in several structured phases to ensure comprehensiveness and accuracy.

In the first phase, two researchers (Li and Wen) independently conducted open coding on five randomly selected interview transcripts using NVivo 15 software, generating initial codes. In the second phase, these two researchers, along with a third researcher (Lin), met to compare their independent coding for one transcript. Through discussion, they developed a preliminary codebook and thematic framework, resolving discrepancies by consensus and refining code definitions. This collaborative calibration ensured initial inter-coder reliability and a shared understanding of the analytic lens. In the third phase, the refined codebook was applied by Li to the remaining transcripts. Throughout this process, the research team held regular meetings to review the evolving themes, discuss analytic memos, and iteratively refine the framework. This involved merging overlapping themes, splitting broad themes into more precise sub-themes, and discarding themes with insufficient or weak supporting data from across the dataset. To validate the final thematic framework and mitigate researcher bias, a critical fourth phase was undertaken. After the research team finalized a draft framework, two independent researchers (Meng and Cao), who were not involved in the initial coding, were invited to perform a blind audit. They were provided with the final thematic framework, codebook, and a subset of anonymized transcripts. Their task was to assess whether the themes accurately and comprehensively represented the data in the provided transcripts. Their feedback was integrated, and all four researchers (Li, Lin, Meng, and Cao) reached a consensus that the thematic framework provided a credible representation of the dataset.

Thematic saturation was assessed using the principle of data saturation (16). We determined that saturation was achieved when analysis of new transcripts yielded no substantial new themes or insights relevant to our research questions, and the properties of existing themes were well-defined and richly illustrated. The results were reported in accordance with the Consolidated Criteria for Reporting Qualitative Research (COREQ) guidelines (17).

### 2.5 Ethical approval and informed consent

The study strictly adhered to the ethical principles of the Helsinki Declaration. Ethical approval was granted by the Shantou University Ethics Committee (approval number: STU2024006001). Prior to the interviews, the research team explained the study objectives and procedures to all participants. Written informed consent was obtained from all participants, who were informed that their participation was voluntary and that they could withdraw at any time. To ensure confidentiality, all personal identifiers were removed, and data were anonymized using unique identification codes.

## 3 Results

The research team conducted 22 in-depth interviews in Guizhou Province in July 2024, including 15 social workers and 7 school administrators. Among the participants, 14 were female (64%) and 8 were male (34%). Participants were from three regions in Guizhou Province (Guiyang City, Xingyi City, and Liping County). The demographic characteristics of participants are summarized in Table 1. All interviews were conducted at the social work organizations. The interviews lasted between 30 and 45 min.

**Table 1:** Demographic characteristics of participants (*N* = 22).

Participant ID	Gender	Age	Education level	Years of service	Location
Social Workers (*n* = 15)					
#2	Female	50	Bachelor’s Degree	10	Guiyang
#3	Female	42	Junior High School	6	Guiyang
#4	Female	40	Secondary Vocational School	7	Guiyang
#5	Male	51	Bachelor’s Degree	22	Guiyang
#7	Female	63	Junior College	40	Guiyang
#8	Male	46	Junior College	10	Guiyang
#9	Female	32	Bachelor’s Degree	8	Guiyang
#10	Female	54	Junior College	14	Guiyang
#13	Male	43	Junior College	17	Guiyang
#15	Female	23	Bachelor’s Degree	1	Guiyang
#19	Female	25	Junior College	1	Xingyi
#21	Female	30	Junior College	5	Xingyi
#22	Female	29	Junior College	4	Xingyi
#6	Female	40	Bachelor’s Degree	11	Liping
#14	Female	30	Bachelor’s Degree	4	Liping
School administrators (*n* = 7)					
#16	Male	36	Bachelor’s Degree	14	Guiyang
#18	Male	51	Bachelor’s Degree	28	Guiyang
#20	Female	30	Bachelor’s Degree	9	Guiyang
#11	Male	39	Bachelor’s Degree	20	Xingyi
#12	Male	50	Bachelor’s Degree	29	Xingyi
#17	Female	43	Bachelor’s Degree	22	Xingyi
#1	Male	41	Bachelor’s Degree	22	Liping

Through thematic analysis of the interview data, this study identified three core themes: Practices of prevention and control, challenges, and pathways for capability enhancement. See Table 2 for details. These are presented in the following sections.

**Table 2:** Themes and sub-themes identified.

Themes	Sub-themes	Categories	Frequency of code
Practices of prevention and control	Diverse prevention and control methods	Health promotion	87
		Multi-sector coordination	45
		Patrol duty systems	16
		Physical protection measures	11
	Intervention effectiveness	Enhanced safety awareness	35
		Reduction in unintentional injury	29
	Resource allocation	Resource acquisition channels	28
		Capacity-building initiatives	11
		Policy support mechanisms	5
Challenges	Internal challenges	Resource constraints	34
		Capacity deficiencies	10
	External challenges	Interdepartmental coordination failures	25
		Developmentally mismatched interventions	24
		Deficient family supervision	14
		Inadequate policy implementation	13
		Insufficient societal recognition and support	12
Pathways for capability enhancement	–	Enhanced resource support	46
		Strengthened cross-sector collaboration	22
		Innovative prevention strategies	9

### 3.1 Practices of prevention and control

#### 3.1.1 Diverse prevention and control methods

Local social organizations in Guizhou Province have adopted diversified methods for the prevention and control of children’s unintentional injuries, primarily manifested in four aspects: health promotion, multi-party collaboration, duty patrols, and physical protection.

In terms of health promotion, social organizations have constructed a multi-dimensional promotion network that includes innovative educational formats, daily safety reminders, and regular home visits. They have broken through the traditional one-way knowledge transfer model of safety education (such as safety lectures) by adopting highly interactive promotional methods like experiential teaching and peer education, making the transmission of safety knowledge more vivid and effective. Furthermore, in collaboration with communities, they utilize various channels including bulletin boards and WeChat groups to reinforce daily safety reminders, integrating safety alerts into daily life. This normalized reminder mechanism ensures the continuous cultivation of safety awareness.

- *“In traffic safety education, we use experiential learning; for example, we conduct demonstrations with actual vehicles like buses and cars. The ‘egg-helmet experiment’ illustrates injury severity from different fall heights, helping children visually understand the importance of protection and discouraging high-altitude object-throwing or climbing.” [#19 Social Worker]*
- *“Our community implements peer education, where older children teach safety to younger children. Due to their similar age, older children are more likely to build trust and empathy with younger ones, which improves the efficiency and acceptance of information delivery.” [#22 Social Worker]*
- *“We conduct regular home visits to inspect the household environment. If hidden dangers are found, we immediately remind the parents and supervise them to make corrections.” [#10 Social Worker]*
- *“We update promotional posters on child unintentional injury prevention daily on the bulletin boards, and simultaneously send these safety tips to the resident WeChat group, reinforcing safety awareness among parents and children.” [#2 Social Worker]*

In terms of multi-party collaboration, the prevention and control of children’s unintentional injuries in Guizhou Province demonstrate the characteristics of multi-sector collaboration. When carrying out related work, local social organizations generally adopt a collaborative model that is government-led and involves multi-agency coordination. By clarifying the roles of each entity and reasonable institutional arrangements, a relatively perfect child safety protection network has been formed. This collaborative mechanism spans both program planning and implementation phases.

- *“The children’s unintentional injury prevention projects carried out by our social workers mainly rely on government support and promote implementation through long-term cooperation with education departments and schools.” [#22 Social Worker]*
- *“Our community has established a protection system involving social workers, street offices, and schools, which jointly carry out round-the-clock patrols and regular home visits. In addition, we have established a rapid response mechanism that coordinates neighborhood committees, parents, and community doctors during emergencies.” [#21 Social worker]*
- *“When the school carries out activities, it will cooperate with the village leaders to help carry out the safety education for parents, due to their community authority and public trust.” [#11 School Administrators]*

For the specific group of ‘left-behind children,’ particularly ‘unaccompanied left-behind children’(defined as children whose parents have both migrated for work for ≥6 months and who have no other guardian), some regions have developed innovative management models. Social organizations, in coordination with schools, have established full-time guardianship model, responsibility system, and grid-based safety management system, which have effectively filled the gap in family supervision and practically ensured the safety of this specific group.

- *“Our school implements a full-time supervision model for unaccompanied left-behind children. That is, they are supervised by teachers at school, and social workers continue to supervise them after school. Concurrently, we implement grid-based management. Every 10 students form a safety group and elect a leader, who is responsible for promptly reporting any safety hazards within the group.” [#18 School Administrator]*
- *“Our school assigns an exclusive responsible teacher to each unaccompanied left-behind child. This teacher is responsible for one-on-one supervision, including conducting regular home visits and screening for safety hazards.” [#1 School Administrator]*

In terms of duty patrols and physical protection, social organizations, in conjunction with sub-district staff and school teachers, form patrol teams to conduct routine inspections of high-risk areas such as bodies of water and transportation corridors. They have also established a 24-h duty patrol mechanism to ensure round-the-clock monitoring of these key areas. For physical protection, schools and community institutions have widely implemented foundational safety modifications. By installing basic protective facilities such as anti-collision strips, protective nets, and non-slip flooring, they have eliminated environmental safety hazards. This prevention and control strategy, which organically combines personnel supervision with environmental modification, has constructed dual protection for child safety.

- *“To address drowning and traffic risks, the community has established a 24-h inspection system, organizing staff to conduct routine patrols of key areas.” [#21 Social Worker]*
- *“All table corners in our organization are fitted with anti-collision strips, and protective nets are installed on key areas like electrical sockets, electric fans, and windows. During home visits, we also focus on inspecting and guiding parents to eliminate domestic safety hazards.” [#4 Social Worker]*
- *“Our kindergarten has anti-slip rubber flooring, all table corners are fitted with anti-collision strips, and the hot water area of the water dispenser is equipped with a protective lock, etc. This effectively ensures the children’s safety while they are at the kindergarten.” [#20 School Administrator]*

#### 3.1.2 Intervention effectiveness

Through the implementation of diversified prevention and control measures, most participants reported that the interventions by social organizations had achieved notable effects on two levels: first, the safety awareness of children and their guardians was significantly enhanced, which translated into specific safety behavior changes; second, the number of children’s unintentional injury incidents in the community decreased, and the severity of injuries was reduced.

The various safety education activities led or participated in by social organizations effectively enhanced the safety awareness of children and parents.

- *“Through safety education, parents now properly store dangerous items (such as medications, cleaning agents) to prevent children from accessing them.” [#14 Social Worker]*
- *“The peer education method, where older children teach younger children, has been particularly effective in improving children’s safety awareness and enhancing safety behaviors.” [#22 Social Worker]*

As key partners of social organizations, school administrators also confirmed the effectiveness of these collaborative interventions, noting that some students had internalized safety knowledge into behavioral habits.

- *“Through the daily ‘5-Minute Safety’ education, students’ safety awareness and self-protection capabilities have been effectively enhanced.” **[#20 School Administrator]***
- *“After long-term safety education, some students have developed safety supervision awareness. When parents violate traffic rules (such as forcing their way across the street during a red light), the students will proactively dissuade their parents, emphasizing, ‘The teacher said we must not go.’” **[#16 School Administrator]***

Furthermore, the joint patrols and physical protection measures in which social organizations participated were significantly effective in reducing actual injury incidents.

- *“Ever since our social work agency and the sub-district office started joint 24-h patrols on dangerous roads and near bodies of water, there have been no more child drowning incidents in our community.” [#21 Social Worker]*
- *“After installing anti-collision strips and cushioned flooring, the risk of injury when children fall has been effectively reduced, and the severity of injuries has also been lowered.” [#4 Social Worker]*

#### 3.1.3 Resource allocation

To support the implementation of the above prevention and control methods, local social organizations in Guizhou have constructed a multi-level, multi-channel resource support system for children’s unintentional injury prevention and control.

In terms of resource acquisition, social organizations have established diversified channels for obtaining resources, including both information resources and funding. Regarding funding, one social worker explained: “The organization’s funding sources, apart from special allocations from the government, include support from charitable foundations and donations from caring individuals” [#7 Social Worker]. In terms of professional information, some social organizations achieve resource sharing by joining industry networks: “Our organization has joined the National Cerebral Palsy Network, which allows us to share high-quality resources within the industry for the prevention of unintentional injuries in disabled children.” [#4 Social Worker].

In terms of professional capacity building, social organizations emphasize the integration of systematic theoretical training with practical experience, building a team equipped with foundational protection knowledge and professional skills. Regarding the care of children with special needs, a social worker stated: “We have received systematic training, including: guidance on correct holding postures, points of caution when assisting with walking, and standardized operations for assisting with drinking and eating.” [#10 Social Worker]. In terms of daily protection, social workers also expressed professional confidence: “The professional knowledge of the organization’s staff regarding child unintentional injury prevention and control is sufficient to meet daily demands. We also have colleagues with nursing backgrounds on the team providing professional support.” [#19 Social Worker].

In terms of policy and institutional guarantees, the prevention and control work for unintentional childhood injuries has received top-down policy support, encompassing specific measures such as policy formulation, special funding support, and the construction of grassroots social work stations. Participants mentioned that the provincial level has issued specific documents requiring the establishment of a long-term mechanism for unintentional childhood injury prevention and control. At the grassroots implementation level, “The government sets aside special funds for child unintentional injury prevention and control projects every year. Both the government and schools strongly support our work.” [#22 Social Worker]. This multi-faceted resource allocation provides a solid foundation of support for the prevention and control efforts.

### 3.2 Challenges

To systematically synthesize and deepen the analysis of the challenges and contextual factors identified, we mapped the findings from the interviews onto a SWOT (Strengths, Weaknesses, Opportunities, Threats) analytical framework (see Table 3). This framework categorizes the operational status of grassroots social organizations, providing a structured overview before detailing specific challenges.

**Table 3:** SWOT analysis of grassroots social organizations in child unintentional injury prevention in Guizhou.

Strengths	Weaknesses
Development of localized, innovative intervention strategies (e.g., experiential education, peer-led advocacy). Established initial cooperation mechanisms with schools and communities Development of grid-based management and full-time guardianship specifically for left-behind children to fill supervision gaps.	Chronic shortages of funding and full-time professional staff limit the coverage and frequency of services. Imperfect and ineffective training system (“Train-the-Trainer” model) leading to professional capacity gaps.

Opportunities	Threats
The implementation of the National Program for Child Development (2021–2030) provides political legitimacy and potential funding channels. Emergence of smart monitoring technologies (e.g., electronic school cards, intelligent early-warning systems) to supplement human supervision. Growing societal attention to child safety and left-behind children issues. Existing multi-sector collaboration models that can be strengthened.	Poor cross-sectoral collaboration and ambiguous responsibility boundaries, leading to governance vacuums. Severe lack of family supervision, especially for left-behind and unaccompanied children. Weak public awareness and a reactive (“neglect before, emphasize after”) social attitude towards prevention.

The following subsections detail the key weaknesses (internal challenges) and threats (external challenges), which constitute the primary challenges, drawing on the interview data presented within this structured understanding.

#### 3.2.1 Internal challenges

The internal challenges for social organizations in Guizhou regarding children’s unintentional injury prevention and control are mainly manifested in two aspects: resource constraints and capacity gaps.

In terms of resource constraints, the dual shortage of human resources and funding severely restricts the in-depth implementation of the prevention and control work by social organizations. Interviewed social workers commonly reported that social work organizations face the predicament of insufficient personnel, funding shortages, and limited space, which makes it difficult to carry out in-depth promotional and educational work. As partners of social organizations, school administrators also confirmed the dilemma that social organizations are limited in cooperation due to their own resources.

- *“Our community organization, due to staffing and funding shortages, has a low frequency and short duration for our in-home promotional visits. The promotional effort is insufficient.” [#8 Social Worker]*
- *“Our organization’s promotional efforts for unintentional injuries are not strong, because we lack sufficient staff.” [#13 Social Worker]*
- *“Because the social work organization has insufficient staff, and its space is also quite small, they can only provide temporary supervision for unaccompanied left-behind children. The level of collaboration is not enough.” [#11 School Administrator]*

In terms of capacity gaps, the professional knowledge and skills of social workers require improvement. Interviewed social workers frankly stated that some social workers have few opportunities for training. The existing training often adopts a ‘secondary transmission’ model, where a few individuals receive external training and then transfer that knowledge to others. This model results in outdated training content, knowledge that is not firmly mastered, and limited coverage, making it difficult to meet practical work needs and directly impacting the effectiveness of safety protection.

- *“Some social workers in our community are still very deficient in knowledge of child unintentional injury prevention and control. They have few learning opportunities, and the learning is not systematic.” [#10 Social Worker]*
- *“Our organization’s staff lack sufficient professional knowledge in injury prevention and control for children with disabilities. Only a few staff members have received systematic training. Most staff learn via ‘secondary learning,’ and their mastery is not solid, which affects the effectiveness of our promotion efforts toward parents.” [#7 Social Worker]*
- *“Our social workers’ first-aid knowledge is truly insufficient. Although we previously organized for some staff to go and learn, and then have them re-teach everyone, this method is not systematic. Furthermore, much of the content is already outdated, and it failed to cover all frontline social workers. This greatly affects our promotional effectiveness.” [#10 Social Worker]*

#### 3.2.2 External challenges

When carrying out their work, social organizations face external challenges from multiple sources, stemming from the collaborative system, the intervention environment, and the service recipients.

Currently, there is a lack of effective coordination among families, communities, schools, and government departments. Ambiguous boundaries of responsibility among these entities create vacuum zones in prevention and control work. There is also a significant discrepancy in the actual implementation of prevention policies. Grassroots work often remains a formality, lacking effective supervision mechanisms and accountability systems, which prevents these measures from being truly effective on the ground.

- *“The reservoir behind our school has no guardrails or warning signs in its dangerous sections. But because it’s not within our jurisdiction, we have no authority to handle it. We can only report it to the local government. However, a drowning incident occurred during this time.” [#17 School Administrator]*
- *“When government departments come to the grassroots to conduct safety promotion, it’s basically just a formality. They take a few work photos and leave. There is a lack of effective supervision, and the policies are not implemented properly.” [#3 Social Worker]*

Secondly, physical safety hazards in the intervention environment are prominent. The hardware facilities of their partners (such as schools) and the surrounding communities have numerous safety hazards, which are beyond the social organizations’ own capacity to rectify.

- *“The guardrails in our school’s hallways are quite low. Students lean against them during breaks, which can easily lead to falls.” [#18 School Administrator]*
- *“The road at our school’s entrance is on a slope. It has no traffic lights and no speed bumps. It’s very easy for traffic accidents to happen.” [#17 School Administrator]*
- *“Our school hallways do not have protective netting installed. Students climb on the railings during breaks, and falls can easily happen.” [#12 School Administrator]*

The systemic lack of family supervision is a major external challenge faced by social organizations in children’s unintentional injury prevention and control. Left-behind children, in particular, face the predicament of absent parental supervision and ineffective grandparental supervision. This is coupled with a generally weak family safety awareness and prominent domestic safety hazards. Economic constraints further exacerbate the supervision dilemma, creating a vicious cycle. The most severe cases involve ‘unaccompanied left-behind children’ who completely lack a parental role and often exhibit dangerous behaviors due to no supervision. This significantly increases the pressure and difficulty of the social organizations’ prevention and control work.

- *“Grandparent guardians are limited by their cognition and physical strength. They mostly adopt a ‘free-range’ model, believing that it is a normal part of growing up for children to get bumped or scraped.” [#14 Social Worker]*
- *“The parents go to work during the day. The child is home alone after school, and then arranges to go play by the pond with a few friends. As a result, a drowning occurred.” [#17 School Administrator]*
- *“There are several children in the fourth or fifth grade whose parents and grandparents have all migrated for work. They live at home all by themselves. These children have to cook for themselves from a young age.” [#14 Social Worker]*
- *“The unaccompanied left-behind children at our school are unsupervised by parents after school and often engage in dangerous behaviors, such as unauthorized touching of 220-volt household circuits or 1,000-volt high-voltage lines. It is extremely dangerous.” [#17 School Administrator]*

There is insufficient public awareness and support for child safety. Staff from social organizations perceived that the current public perception of child safety follows a passive model of ‘neglect beforehand, emphasis afterward’, making it difficult to establish long-term protection mechanisms. The safety issues of children with special needs have not received the attention they deserve. There is a lack of public awareness regarding assistive devices for children with disabilities (such as hearing aids), making it difficult to foster a social atmosphere of collective protection. This also increases the resistance social organizations face in carrying out their work.

- *“We usually do not pay enough attention to unintentional injuries. They are only taken seriously when an incident occurs, but they are quickly forgotten afterward.” [#2 Social Worker]*
- *“Accessibility facilities are merely symbolic. Problems like tactile paving being occupied and footbridges lacking accessible ramps are widespread.” [#9 Social Worker]*

### 3.3 Pathways for capability enhancement

Participants believed that the current prevention work is constrained by the internal and external challenges identified above. Accordingly, they proposed three key pathways for enhancement: enhanced resource support, strengthened cross-sector collaboration, and innovative prevention strategies.

To address the internal challenges of funding shortages and capacity gaps, participants emphasized the urgency of enhanced resource support. Regarding funding, social workers suggested broadening revenue channels beyond government grants to include corporate and social donations. Regarding the capacity limitations of the “secondary transmission” training model, participants called for systematic, expert-led professional training to replace the current approach and ensure frontline competence.

- *“Social work organizations are limited by funding shortages, so service coverage is limited. There are over 300 children in the community, but we can currently only serve about 100. To improve this situation, the key is to secure more financial support. Only with sufficient funding can we develop higher-quality activity content.” [#22 Social Worker].*
- *“There is a need to increase the opportunities and frequency of professional training for social workers; this is key to improving the quality of safety education.” [#10 Social Worker].*

Targeting the external challenge of “regulatory vacuums” and ambiguous responsibilities, participants widely advocated for strengthened cross-sector collaboration. They believed that establishing a government-led joint prevention mechanism is essential to effectively integrate resources from communities, schools, and families, thereby resolving the “responsibility without power” predicament.

- *“Child safety prevention and control should be led by the government, integrating the efforts of the community, schools, and other parties to form a joint prevention and control work structure.” [#13 Social Worker].*

In response to the challenge of supervision deficits for left-behind children and the low engagement of traditional education, participants suggested innovative prevention strategies. This includes upgrading educational forms to be more interactive and, crucially, applying smart technologies (e.g., electronic school cards) to supplement human supervision blind spots.

- *“Existing safety education activities lack innovation in both form and content, making it difficult to maintain children’s interest in participation. We must put more effort into innovating safety education activities.” [#19 Social Worker].*
- *“We are cooperating with a telecommunications company to develop a smart early-warning system that monitors dangerous areas in real-time via electronic school cards.” [#1 School Administrator]*

## 4 Discussion

This study employed semi-structured in-depth interviews to explore the practices, challenges, and enhancement pathways of grassroots social organizations in Guizhou Province regarding child unintentional injury prevention.

Regarding practices, this study found that grassroots social organizations have developed effective localized intervention strategies. By employing experiential interactive teaching and peer education, they have successfully internalized safety knowledge into children’s behavioral habits. This successful practice substantiates the Theory of Planned Behavior (18) and Social Learning Theory (19), indicating that sustained promotional education and peer demonstration can translate cognition into behavioral changes. Furthermore, the “Government-School-Community” collaboration has constructed a preliminary safety net. Specifically, to address the severe supervision deficits for left-behind children, local actors have innovated “grid-based management” and “full-time guardianship” models. These localized efforts demonstrate that even in resource-constrained settings, grassroots actors can develop adaptive solutions to mitigate environmental risks.

Despite these promising localized practices, our analysis, structured through a SWOT framework (Table 3), reveals that their sustainability is precarious. Grassroots organizations are trapped in a dilemma where internal weaknesses limit their ability to respond to systemic external threats. A critical internal weakness is the “secondary transmission” training model (Train-the-Trainer) (20). Although the Train-the-Trainer model is widely adopted in resource-scarce regions due to its low cost, its inherent flaws are clearly exposed in this study. Its flaw is far more than just knowledge decay; it is knowledge distortion. This means that even if the initial training is high-quality, after being relayed through multiple layers, the knowledge obtained by frontline social workers is already fragmented. It cannot effectively guide their promotional efforts toward parents or their response to emergencies, thereby severely weakening the intervention’s effectiveness. This predicament is similar to the grassroots training dilemmas found in other developing countries, such as India (21). Regarding resource allocation, while Guizhou has constructed a multi-level support system similar to ‘The Child Accident Prevention Trust’ in the UK (22), the chronic instability of funding remains a weakness that restricts service continuity.

These internal fragilities are exacerbated by severe external threats, primarily the structural deficit of family supervision. Economic pressures and the household registration (hukou) system (23) jointly shape this dilemma: parents migrate for work, but children are restricted by their hukou and cannot relocate, leading to single-parent, intergenerational, or even “unaccompanied” living situations (24, 25). This phenomenon parallels child injury patterns caused by seasonal migration in other developing countries like India (26), highlighting the shared challenges of rapid urbanization. Furthermore, organizations face the threat of “responsibility without power” due to collaboration failures. Ambiguous boundaries lead to regulatory vacuums (e.g., drowning incidents in boundary waters). Additionally, the universal challenge of child developmental characteristics, such as young children’s impulsiveness (27), requires interventions to be highly age-appropriate, yet current strategies often lack this precision.

The findings of this study have significant implications for public health policy and practice. First, this study fills a critical evidence gap regarding injury prevention in western, economically underdeveloped regions of China. Unlike previous studies that predominantly focused on developed eastern regions or medical-led models (10–12), this research exposes the unique “double jeopardy” faced by grassroots organizations in the west: the convergence of high environmental risks (left-behind children) and low institutional capacity (funding/training deficits). Second, the study provides an operational evidence base for implementing the China National Program for Child Development (2021–2030) in resource-scarce settings. It suggests that simply setting top-down targets is insufficient; success requires building a multi-source resource support network and transitioning from cascade training to systematic professional capacity building. Finally, the study highlights the necessity of structural interventions over mere education. The proposed pathways, such as government-led cross-sectoral mechanisms and the integration of smart monitoring technologies, offer a blueprint for optimizing the governance of children’s safety at the grassroots level, transforming the system from passive response to active prevention.

Implementing these pathways in less developed regions such as Guizhou requires pragmatic adaptation. A phased approach is recommended, first prioritizing the strengthening of existing core partnerships (such as between social organizations and schools) and leveraging available resources (such as small grants and community fundraising). The proposed “government-led cooperation” may in practice mean formalizing existing local networks rather than creating new structures. Similarly, innovative strategies should prioritize low-cost, high-impact tools such as social media in education. The insight to be drawn for similar areas is to apply the principles of cooperation, diversified resources, and tailored interventions to incremental actions based on the specific circumstances of local social capital.

This study has certain limitations. First, as a qualitative study, its conclusions are primarily based on the subjective statements of participants and the interpretive analysis of the researchers, which may introduce interpretation bias. Future research should integrate quantitative data to strengthen the argumentation. Second, regarding the sample distribution, a larger proportion of participants were from Guiyang City (the provincial capital). This distribution reflects the objective reality of the uneven development of social organizations in Western China, where resources and professional agencies are highly concentrated in provincial capitals. While this may introduce a geographical bias, the inclusion of participants from Liping and Xingyi ensured that the distinct challenges of grassroots, resource-constrained settings were captured. Future research should strive for a more balanced sample across urban and rural areas within underdeveloped regions to enhance the generalizability of findings. Third, the study sample only covered parts of Guizhou Province. Limited by regional culture and socioeconomic factors, the generalizability of the findings requires further verification. Future research could be expanded to other regions for comparative studies.

## 5 Conclusion

By conducting an in-depth analysis of the practices of local social organizations in Guizhou Province in children’s unintentional injury prevention and control, this study revealed both their innovative potential and the systemic challenges they face. The study found that social organizations, through localized methods such as experiential education and peer education, combined with a government-school-community collaborative mechanism, effectively enhanced children’s safety awareness and injury prevention outcomes. However, internal constraints such as resource scarcity and insufficient professional capacity, as well as external dilemmas including inadequate cross-sectoral collaboration and weak family supervision, continue to restrict the effectiveness of prevention and control. To address these challenges, three evidence-based recommendations emerge: First, constructing a multi-source resource support network to increase financial and human resource investment. Second, improving the government-led cross-sectoral collaboration mechanism to clarify the division of responsibilities. Third, developing intervention strategies adapted to children’s cognitive characteristics and innovating technological applications. These insights, based on the practices in Guizhou Province, not only provide an evidence base for optimizing the unintentional childhood injury prevention and control system in economically underdeveloped regions—with particular practical value for improving protection mechanisms for specific groups like left-behind children—but also offer an operational reference pathway for the implementation of the injury prevention and control objectives set forth in the China National Program for Child Development (2021–2030) in similar, underdeveloped areas.

## Data Availability

The qualitative data from this study is available to bona fide researchers upon request from the corresponding author. Access to the data is controlled to protect the privacy of participants and the community.
