# Supplementary material for: Exploring the practices, challenges, and pathways for enhancement of grassroots social organizations in preventing child unintentional injuries: a qualitative study in Guizhou, China
Source: Front Public Health. 2026 Jan 29;14:1760634. doi: 10.3389/fpubh.2026.1760634 (PMC12894380; doi:10.3389/fpubh.2026.1760634)
Supplement: Supplementary file 1 [file Table_1.DOCX]

**Interview Guides**

**Exploring the Practices, Challenges, and Pathways for Enhancement of Grassroots Social Organizations in Preventing Child Unintentional Injuries: A Qualitative Study in Guizhou, China**

### ****I. In-Depth Interviews: School Administrators****

****1. The cognition, prevention and control status of children's unintentional injury****
(1) What types of child unintentional injuries are you aware of?
(2) What specific prevention measures or policies has your school implemented?
(3) How are these measures executed, and what is their implementation frequency?

****2. Intervention Effectiveness Evaluation****
(1) How effective are these measures? Is there supporting data?
(2) Which interventions have been most effective? Provide examples or data.

****3. Current State of Social Organizations’ Prevention Capabilities****
(1) Which local social organizations participate in children’s injury prevention? What measures do they use, and how effective are they?
(2) How do these organizations collaborate with schools? What outcomes have resulted?
(3) What unique roles can social organizations play in prevention work?

****4. Difficulties and Challenges****
(1) What challenges has your school faced in implementing prevention measures?
(2) What are the root causes of these challenges?
(3) How is the school currently addressing these challenges?

****5. Innovations and Practices****
(1) In the prevention and control work, what are your school’s innovative approaches or successful cases?
(2) How were these innovations developed, and what external support was received?
(3) What impact have these innovations had on prevention capabilities?

****6. Multi-sectoral cooperation and linkage****
(1) How should schools, government, families, and communities collaborate?
(2) What current collaboration channels exist between schools and government/social organizations/families?
(3) How can cross-sector collaboration be strengthened?

****7. Gap Assessment****
(1) What are the limitations of current interventions in your school? How do they affect outcomes? What improvements do you suggest?
(2) Do teachers have sufficient expertise in children’s injury prevention? What skills need urgent enhancement?

****8. Conclusions and Recommendations****
(1) What aspects require urgent reinforcement in child injury prevention?
(2) What specific recommendations in child injury prevention do you have for Guizhou’s context?
(3) Is there anything else you would like to add about children's unintentional injury prevention and control?

### ****II. In-Depth Interviews: Social Work Practitioners****

****1. The cognition, prevention and control status of children's unintentional injury****
(1) What types of child unintentional injuries are you aware of?
(2) What prevention measures has your organization implemented?
(3) How are these measures deployed in communities? What is their frequency and coverage?

****2. Intervention Effectiveness Evaluation****
(1) How effective are these measures?
(2) Which interventions show the highest efficacy? Provide examples or data.
(3) What unique role do you think social organizations play in the prevention and control of children's unintentional injury?

****3. Difficulties and Challenges****
(1) What major challenges has your organization encountered in carrying out the work of children's unintentional injury prevention and control?
(2) What are the underlying causes?
(3) How is your organization addressing these challenges?

****4. Innovations and Practices****
(1) In the prevention and control work, what are your organization’s innovative approaches or successful cases?
(2) How were these innovations created, and what external support was received?
(3) What impact have they had on prevention capabilities?

****5. Multi-sectoral cooperation and linkage****
(1) How should communities, schools, government, and families collaborate?
(2) What collaboration channels exist between social organizations and schools/government/families?
(3) How can we further strengthen the collaboration of these entities in the prevention and control work?

****6. Gap Assessment****
(1) What are the limitations of your organization’s interventions? How do they affect outcomes? What improvements do you suggest?
(2) Do social workers have sufficient expertise in children’s injury prevention? What skills need urgent enhancement?

****7. Conclusions and Recommendations****

(1) What aspects require urgent reinforcement in child injury prevention?
(2) What specific recommendations in child injury prevention do you have for Guizhou’s context?
(3) Is there anything else you would like to add about children's unintentional injury prevention and control?
